# The plant vampire diaries: a historic perspective on *Cuscuta* research

**DOI:** 10.1093/jxb/erad082

**Published:** 2023-03-08

**Authors:** Maleen Hartenstein, Markus Albert, Kirsten Krause

**Affiliations:** Department of Biology, Molecular Plant Physiology, Friedrich-Alexander-Universität Erlangen-Nürnberg, Staudtstr. 5, D-91058 Erlangen, Germany; Department of Biology, Molecular Plant Physiology, Friedrich-Alexander-Universität Erlangen-Nürnberg, Staudtstr. 5, D-91058 Erlangen, Germany; Department of Arctic and Marine Biology, UiT The Arctic University of Norway, Framstredet 39, 9019 Tromsø, Norway; MPI of Molecular Plant Physiology, Germany

**Keywords:** *Cuscuta*, dodder, haustorium, interspecific cell–cell connections, parasitic plant, parasitic plant genome evolution, plant–plant interaction, plant resistance

## Abstract

The angiosperm genus *Cuscuta* lives as an almost achlorophyllous root- and leafless holoparasite and has therefore occupied scientists for more than a century. The ‘evolution’ of *Cuscuta* research started with early studies that established the phylogenetic framework for this unusual genus. It continued to produce groundbreaking cytological, morphological, and physiological insight throughout the second half of the 20th century and culminated in the last two decades in exciting discoveries regarding the molecular basis of *Cuscuta* parasitism that were facilitated by the modern ‘omics’ tools and traceable fluorescent marker technologies of the 21st century. This review will show how present activities are inspired by those past breakthroughs. It will describe significant milestones and recurring themes of *Cuscuta* research and connect these to the remaining as well as newly evolving questions and future directions in this research field that is expected to sustain its strong growth in the future.

## Teaser

A century of research on the parasitic angiosperm genus *Cuscuta*has contributed to our understanding of numerous plant and cell biological aspects.

## Introduction

Plants that procure some or all their nutrients from other living plants are defined as parasitic (Kuijt, 1969; Heide-Jørgensen, 2008). The phenomenon of plants that have given up an autotrophic lifestyle to turn to an almost predatory-like lifestyle has bestowed a fair bit of mysticism upon them that is still reflected in some of the common names that people have given parasitic plants: witchweeds (genus *Striga*), devils thread or angel hair (genus *Cuscuta*), ghost plant (genus *Monotropa*), corpse flower (genus *Rafflesia*), or stemsuckers (genus *Pilostyles*). To some degree, these names reflect the aberrant habitus or nutrition strategy of these parasites that no longer need to maintain their own photosynthetic machinery or an elaborate root system.

**Fig. 1.**
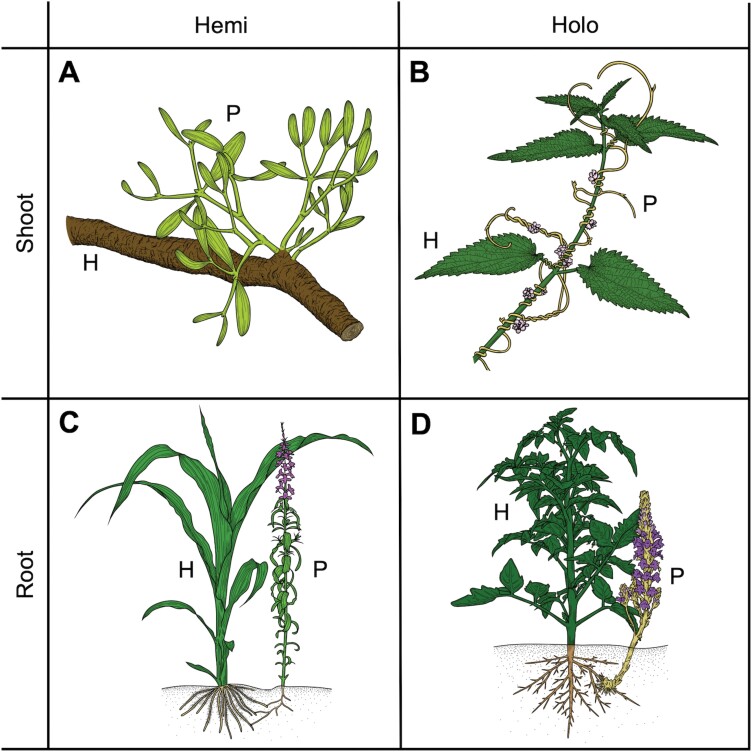
Representative drawings of four different parasitic plants that reflect the different parasitic strategies. (A) For hemiparasitic shoot parasites, *Viscum album* infecting *Pinus sylvestris* is depicted. (B) For holoparasitic shoot parasites, *Cuscuta europaea* infecting *Urtica dioica* is shown. (C) For hemiparasitic root parasites, *Striga hermonthica* infecting *Zea mays* is displayed. (D) For holoparasitic root parasites, *Phelipanche ramosa* infecting *Solanum lycopersicum* is pictured.

Parasitism has manifested itself in >4750 species from >12 independent lineages within the angiosperms (Nickrent, 2020). Well-known parasites are the mistletoes and the dodders that attach to the aerial parts of other plants such as branches or stems of herbs, shrubs, and trees (Fig. 1). These are classified as shoot parasites, as opposed to root parasites like *Striga* or *Phelipanche* that establish underground connections with their hosts (Fig. 1). Other classifications are based on the extent of host dependency or the extent of the ability to carry out photosynthesis, leading to classifications of holo- versus hemiparasites and obligate versus facultative parasites, respectively (Fig. 1). Furthermore, some parasites are highly host specific while others can infect a broad range of angiosperms without inducing a resistance reaction. 

**Fig. 2.**
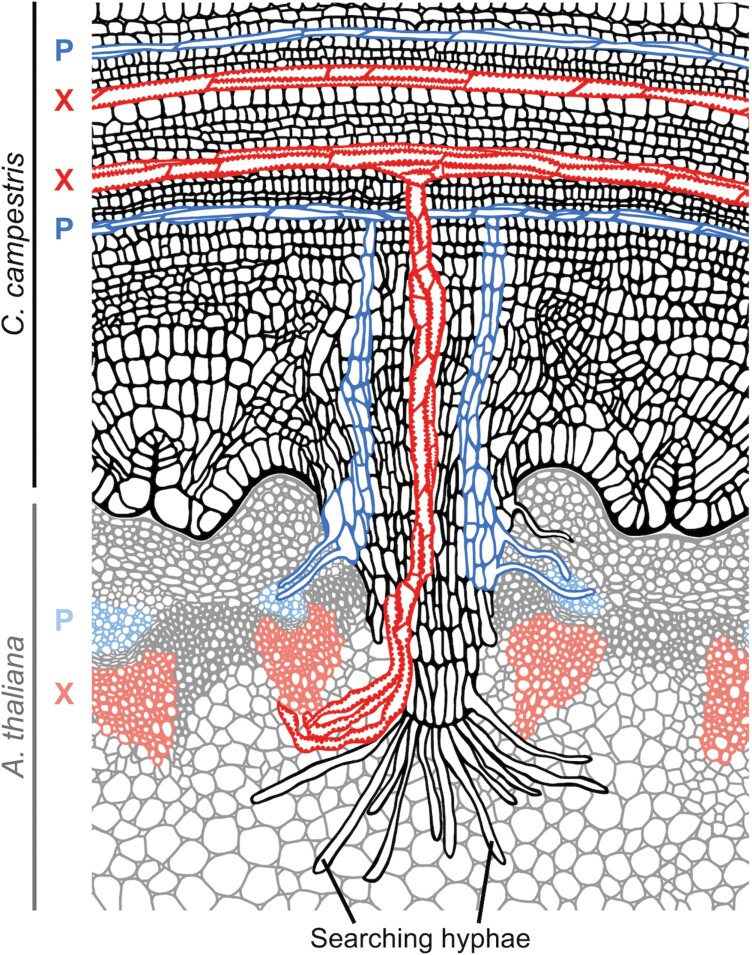
Schematic drawing of the haustorium of *C. campestris* (black outlines) infecting *A. thaliana* (gray outlines). The vascular tissue of both plants is highlighted in blue (P, phloem) and in red (X, xylem) (darker color for the parasite and lighter color for the host).

Despite an obvious variability in the degree of specialization, all parasitic plants have one trait in common that can be denoted their ‘signature organ’: the haustorium (Yoshida *et al.*, 2016) (Fig. 2). The term haustorium originates from the Latin word *haurire* and means to drink, to absorb, to engulf, or to penetrate, depending on the context. The term could not reflect this multicellular organ’s role better, as it enables the parasites to connect physically and physiologically to the host and to extract all essential nutrients. Originally, the term haustorium [sometimes also called ‘haustorium proper’ (Kuijt, 1969)] was often exclusively used to refer to the endophytic tissue of the parasite developing within the host, but its usage was already broadened in the early 1990s by Heide-Jørgensen who in his definition of the haustorium of *Cassytha pubescens* referred to both the attachment structures and the endophytic tissue (Heide-Jørgensen, 1991). A wider definition has become more and more popular in recent literature. However, it is important to stress that the term ‘haustorium’ should not be confused with the term ‘infection site’, which by definition encompasses the adjacent tissue of the host. A reflected, uniform use of the term could also avoid confusion in anatomical and molecular studies, where more precise terms such as ‘upper haustorium’, ‘pre-haustorium’, and ‘adhesive disk’ for exophytic parts and ‘haustorium proper’ for the mature endophytic part of the parasitic infection organ are preferable.

*Cuscuta* spp. (also known as dodders) represent the only parasitic genus in the family of Convolvulaceae (order Solanales). The genus is relatively large, with ~200 species (Nickrent, 2020). *Cuscuta* consists of threadlike and leafless twining stems of 1–3 mm in diameter that can infect all aerial parts of soft herbs but also woody shrubs and even trees (Dawson *et al.*, 1994). Together with the broomrapes and mistletoes, dodders are among the best-researched model organisms for parasitic plants, primarily because their unique appearance, world-wide distribution, and negative impact on agricultural yields has created a substantial amount of interest in them. *Cuscuta* species have also been attributed antibacterial, antioxidant, anti-inflammatory, or hepatoprotective effects, to name a few of the pharmacological applications that have been reported, and that are exploited in traditional Asian medicine (Noureen *et al.*, 2019).

### Early research on *Cuscuta*

Mention of parasitic plants in the literature dates back as far as ancient Greece. Theophrastus, Aristotle’s student (373–287 BC), mentioned *Cuscuta* and other parasitic plants as early as 300 BC in his scripture *Enquiry into Plants*, although the names he used do not correspond to the species names that are common today. Theophrastus described a plant he called *Orobanche*, whose characteristics can be clearly attributed to our present-day *Cuscuta.* A plant that by Theophrastus’ description closely resembles the root parasite we know today as *Orobanche* was referred to by him as *Haimodoron* (Costea and Tardif, 2004).

**Fig. 3.**
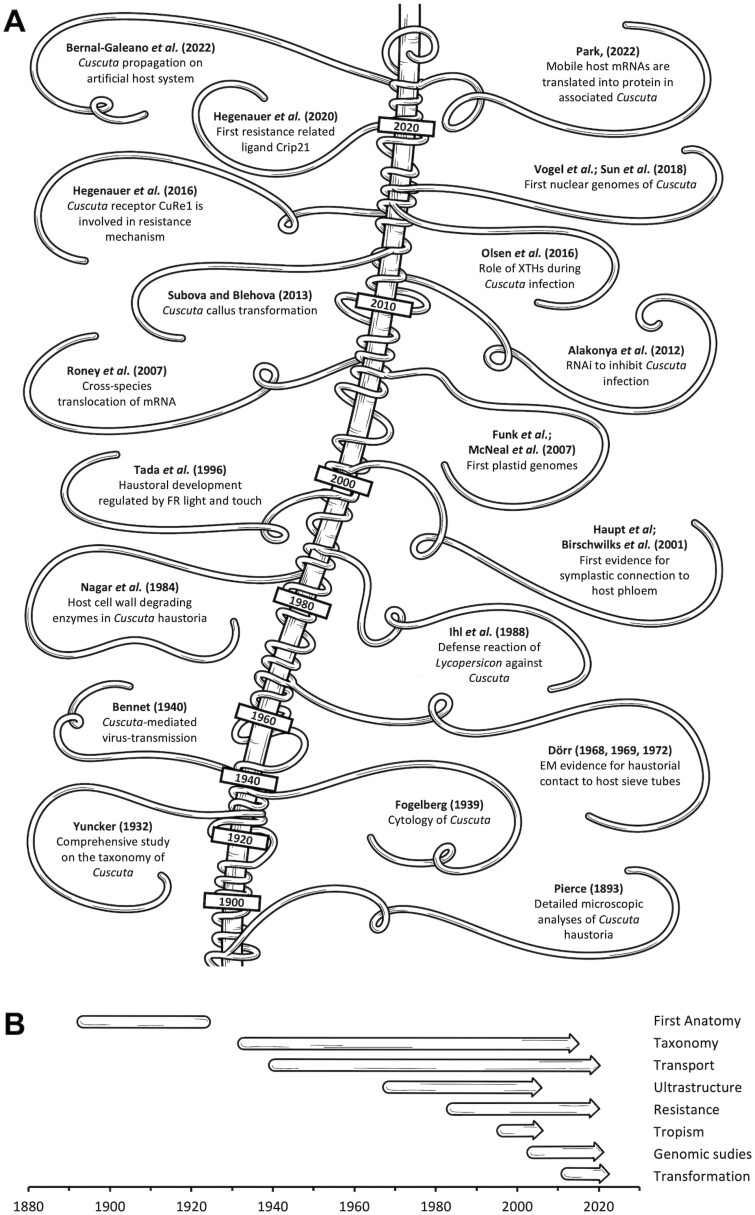
(A) Timeline showing milestones in *Cuscuta* research over the last century. The central time axis is represented by a stylistic host-entwining shoot, while side shoots represent a selection of milestones for the topics detailed in the sections of this review. (B) Main areas of *Cuscuta* research and their major periods of activity, as reported in this review. Arrowheads indicate that activity still continues in multiple fields.

Up until a hundred years ago, the literature largely addressed the occurrence of *Cuscuta*s, their host specificity (or lack thereof), as well as the systematics of this genus. A dominating authority on that subject was Truman G. Yuncker (1891–1964) whose work succeeded that of the famous German–American botanist George Engelman (1809–1884). Engelmann proposed to arrange *Cuscuta* species into three subgenera (Engelmann, 1859), *Grammica*, *Monogyna*, and *Cuscuta*, and Yuncker used these to group 158 *Cuscuta* species in his pivotal study from 1932 (Yuncker, 1932) (Fig. 3). The phylogenetic classification was based mostly on visual taxonomic characteristics from the inflorescences and fruits. This classification has provided a valuable taxonomic framework for many decades and is still valid, although more recently it was suggested that a small number of African species be separated from the subgenus *Cuscuta* and placed in their own subgenus, *Pachystigma*, as confirmed in recent refinements of the phylogenetic framework by Costea *et al.* (2015). Among the other revelations in the newer literature is the observation of extensive hybridization events and reticulate evolution within the large subgenus *Grammica*. The exact number of these events is not clear, and current estimates are probably too low (Stefanovic and Costea, 2008).

At around the same time as Yuncker performed his extensive studies, the first cytological studies were published by Fogelberg (1938) who observed unusually large variations of chromosome sizes and numbers within this genus. These differences were later confirmed by modern flow cytometric and genomic methods and showed >100-fold variations of genome sizes between *Cuscuta* species (Neumann *et al.*, 2021). It is currently assumed that these differences reflect inflations of repetitive regions on the chromosomes.

### Morphological and physiological signatures of parasitism in *Cuscuta*

One of the most conspicuous consequences of the heterotrophic lifestyle of *Cuscuta* at the morphological level is the evolution of the multicellular parasitic organ termed the haustorium (Fig. 2). The structure and development of haustoria was, accordingly, a recurrent theme in *Cuscuta* research (Yoshida *et al.*, 2016). Some of the first detailed microscopic observations of *Cuscuta* haustoria were documented by Peirce (1893) and Thoday (1911). Peirce’s work contained detailed descriptions of phloem and xylem bundles in haustoria of several *Cuscuta* species and he compared haustorium formation with lateral root development. Thoday extended the ideas of Pierce and referred to the haustorium as an adventitious root. She contributed numerous detailed drawings of the haustorial cells, especially the haustorial vascular tissue. Thoday’s and Peirce’s work was challenged by Thomson (1925) who disagreed with the presence of phloem in *Cuscuta* haustoria, and it was not until Dörr, Kollmann, and co-workers published their series of excellent electron microscopy studies (Dörr, 1968, 1969, 1972, 1990; Israel *et al.*, 1980) that this question was settled and Thomson was proved wrong. The notion that cells with sieve tube characteristics are formed in the *Cuscuta* haustoria after successful infection on a susceptible host is no longer disputed. These vascular cells are connected to the host’s sieve tube system through specialized phloic hyphae (Fig. 2). Sieve element–companion cell (SE/CC) complexes in haustoria showed no significant structural differences compared with usual angiosperm phloem cells, but their origin is different; instead of emerging from cambial tissue which is the case in non-parasitic plants, haustorial SE/CC complexes originate from ordinary parenchyma cells (Dörr, 1990; Vaughn, 2006).

Thomson (1925) also noticed that *Cuscuta* spp. have no leaves, lack stomata, and contain only small amounts of chlorophyll. He concluded correctly that *Cuscuta* is most probably unable to photosynthesize sufficiently to cover its demand for carbohydrates. This fact was controversially discussed for the following two decades, as presented by Yuncker (1943). Yuncker summarized how various authors have disagreed on the amount of chlorophyll, the presence or absence of stomata, and the ability to photosynthesize. In his own studies, he presented that at least 11 *Cuscuta* species possess stomata, though in reduced numbers (Yuncker, 1943). Finally, though much later, the work done by Clayson and colleagues provided an explanation for the general disagreement about the presence or absence of stomata in different *Cuscuta* species. Using SEM, they demonstrated that the number and occurrence of stomata vary between species but also between different types of stems within a species (Clayson *et al.*, 2014).

Measurements of photosynthesis and CO_2_ fixation have contributed significantly to the understanding of the hetero-trophic lifestyle of *Cuscuta*. Pattee’s dissertation included photosynthesis studies and ^14^C fixation experiments of *Cuscuta* spp. shoots. The results indicated that the photosynthesis rates of *Cuscuta* spp. only added to an insignificant degree to the nutrient uptake from the host plant (Pattee, 1960). Photosynthetic activity of dodders was also under investigation a few decades later when the work performed by Machado and Zetsche (1990) and van der Kooij *et al.* (2000) confirmed that none of the *Cuscuta* species investigated was able to reach the CO_2_ compensation point, not even species such as *C. reflexa* that appear green. Thus, it can be stated that *Cuscuta* is not able to support any net growth based on its own CO_2_ fixation. This is consistent with the fine structure of the parasite’s chloroplasts. Instead of displaying the typical organization of thylakoids into grana stacks and stroma thylakoids that is symptomatic for autotrophic plants, *Cuscuta* plastids are often less complex with significantly fewer thylakoids and no or negligible amounts of grana stacks. Some variation was observed between different *Cuscuta* species (van der Kooij *et al.*, 2000), with *C. reflexa* exhibiting more and better organized thylakoids than *C. campestris*, while *C. odorata* did not have any thylakoids at all. The lack of structure, therefore, follows roughly the same gradient that was observed for the chlorophyll content of these species. It was hypothesized that *Cuscuta* plastids may have a predominant function in synthesizing and storing starch and lipids, rather than acting as compartments for photosynthesis (Machado and Zetsche, 1990).

### Plastome and genome evolution

The unarguably most characteristic feature of parasitic plants is their transition from autotrophy to a heterotrophic lifestyle. As a result of a reduced evolutionary pressure on maintaining the photosynthetic apparatus, this coincided with changes in the genomes that encode the subunits of the photosynthetic machinery, most evidently in the plastid genomes. Hence the plastid genomes of parasitic plants have attracted a lot of attention since the turn of the century when commercial sequence analyzers based on non-radioactive technology, the predecessors of high-throughput sequencers, became widely popular.

Plastid genomes from autotrophic plants are typically highly conserved in gene content and gene order, and are transcribed and translated in a bacteria-like style from polycistronic operons, necessitating post-transcriptional processing to yield functional monocistronic mRNAs, rRNAs, or tRNAs. Since plastid genomes are dependent on two types of polymerases for their expression, one for housekeeping genes and the other for the components of the photosynthetic machinery, the focus during the first decade of this century was two-fold: on the plastid coding capacity and on the machinery involved in plastid gene expression (Berg et al., 2003, 2004; Krause *et al.*, 2003; Revill *et al.*, 2005; Stefanovic and Olmstead, 2005; Funk *et al.*, 2007; McNeal *et al.*, 2007) (Fig. 3). These investigations revealed progressive plastid genome reductions that are most pronounced in species where plastid functions are severely reduced (Krause, 2011). In addition to the losses of all genes for some multisubunit complexes such as the NADH dehydrogenase complex, a ‘ripple’ or ‘domino’ effect was observed on gene regulatory pathways. Examples are the loss of the plastid-encoded polymerase that is accompanied by a loss of the promoter sequences recognized by it, or the loss of an intron maturase and a concomitant loss of plastid introns, and last but not least the loss of some ribosomal proteins and tRNAs (Krause, 2011). Interestingly, several of the lost genes cannot be deleted in autotrophic plants without losing viability (Krause and Scharff, 2014).

Following the reduction in costs for whole-genome sequencing, the nuclear genomes of two *Cuscuta* species were published recently (Sun *et al.*, 2018; Vogel *et al.*, 2018) (Fig. 3). Their thorough scrutiny revealed that the majority of the lost plastid genes were not transferred to the nucleus but lost entirely. What is more, a significant number of nuclear-encoded genes involved in the same processes were also lost (Sun *et al.*, 2018; Vogel *et al.*, 2018). An impressive example is the loss of nuclear-encoded sigma factors and other RNA-binding proteins from the nuclear genome that interact with the plastid-encoded RNA polymerase or with plastid introns (Schwacke *et al.*, 2019). Unsurprisingly, this indicates close feedback loops between the genomes of this plant.

A separate topic that promises more discoveries once the number of completely sequenced plant genomes rises further is the observation of horizontal gene transfers (HGTs). Vogel *et al.* (2018) reported a high confidence set of 64 genes that were acquired by *C. campestris* from other plants by HGT, while Yang *et al.* (2019) identified 108 probably functional HGTs in the same species (some of those shared with root parasitic Orobanchaceae) in addition to 42 host transposon-derived transfers. A horizontally transferred miRNA in the nuclear genome of *C. campestris* (Zangishei *et al.*, 2022) as well as a functional HGT into the mitochondrial genome were recently described (Lin *et al.*, 2022). Once both quality and quantity of the genomic resources in *Cuscuta* is improved, our understanding of the mechanisms as well as the functional significance of HGT will be increased and should reveal the fundamental evolutionary processes underlying the transition to parasitism.

### Transport processes between host and *Cuscuta*

The use of *Cuscuta* as a ‘living bridge’ to infect recalcitrant plants with viruses for research purposes is widely practised today and is based on the practical knowledge that the parasite acts as a vector for a variety of plant diseases (Bhat and Rao, 2020). Modern investigations considered interspecific plasmodesmata as a unique trait in this interaction as summarized by Fischer *et al.* (2021). However, the question of how transport between host and parasite is accomplished has been discussed for a long time. Based on histological studies, several researchers had concluded early on that water and nutrient transfer must occur through the close contact between parasitic hyphae and host xylem and phloem, respectively (Peirce, 1893; Thoday, 1911). However, the exact mechanism of this exchange was not yet clear. From the 1930s onwards, it was intensely debated whether the contact between host and parasite is established via plasmodesmata, as first proposed by Schuhmacher and Halbsguth (1938). Bennet pursued the same idea and investigated whether the transmission of plant viruses from an infected to a healthy host plant via dodder is successful. He demonstrated that dodder can transmit viruses and recognized the possibility of using *Cuscuta* spp. to study plant viruses (Bennett, 1940). Since virus transmission from cell to cell via plasmodesmata was an acknowledged pathway, Bennet concluded that the virus transmission provided circumstantial evidence for the existence of interspecific plasmodesmata at the interface, a claim that he thought he had substantiated by identifying structures resembling plasmodesmata between haustorium cells and host parenchyma cells in free-hand sections of the infection sites. In contrast, plasmodesmata between haustorial cells and the phloem cells of the host were not identified with certainty (Bennett, 1944). Pattee (1960) attempted to further interpret Bennet’s results by adding his own findings. He demonstrated that ^14^C-labeled compounds fixed by alfalfa infected with dodder accumulated in the attached dodder. He compared the movement of ^14^C-labeled compounds from host to parasite with Bennet’s findings regarding virus transmission. The underlying mechanism of movement from host phloem into haustorial cells was not evident in either case (Bennett, 1944; Pattee, 1960). A few years later, Dörr published detailed electron microscopic pictures of the close contact between *Cuscuta* hyphae and host cells. Just like Bennett (1944), she identified numerous plasmodesmata between hyphae and host parenchyma, but none between hyphae and phloem tissue. Hyphae that establish a close connection with sieve tubes were named contact hyphae because they develop a special absorbing structure. Contact hyphae enlarge their surface by 6–20 times with membrane protuberances facing the host sieve tube. Dörr therefore assumed that the host phloem nutrients are first released into the cell wall and then taken up from the apoplast by the contact hyphae. The surface enlargement of the contact hyphae promotes the uptake (Dörr, 1969, 1972) (Fig. 3).

Contrary to these data, Haupt and colleagues reported results implying the presence of plasmodesmata between contact hyphae and host phloem (Haupt *et al.*, 2001) (Fig. 3). Phloem-mobile green fluorescent protein (GFP) expressed in transgenic tobacco as well as other fluorophores moved from infected transgenic hosts into *Cuscuta*, indicating a symplasmic pathway for macromolecules between host and parasite (Haupt *et al.*, 2001). Transmission rates of different radioactively labeled compounds, including sucrose, amino acids, phytohormones, and xenobiotics, were also measured and corroborated the assumption of a non-selective ‘highway’ for metabolites between the two plants (Birschwilks *et al.*, 2006). All these results provide strong evidence for an open symplasmic connection between *Cuscuta* spp. and the host phloem (Birschwilks *et al.*, 2006). However, so far only Lee (2009) was able to identify plasmodesmata between *Cuscuta japonica* hyphae and the sieve elements of the host plant *Impatiens balsamina* in electron microscopic pictures. The lack of further direct evidence was discussed recently (Fischer *et al.*, 2021).

Along with the emerging subject of long-distance transport of RNAs and the evidence for interspecific plasmodesmata, another focus in *Cuscuta* research was on the possible exchange of RNAs between host and parasite. Several research articles and reviews were published that demonstrate the transmission of host RNAs and their processing in *Cuscuta* spp. (Roney *et al.*, 2007; Leblanc et al., 2012, 2013; Kim *et al.*, 2014; Kim and Westwood, 2015; Park *et al.*, 2021). Besides mRNAs, si RNAs and miRNAs were also shown to cross the host–parasite border in both directions. Although it was initially debated whether RNAs were functional after their transport, it was recently shown that parasite miRNAs can target host mRNAs, and host RNAs can be translated in the parasite into proteins, suggesting that they serve a purpose in the respective interaction partner (Shahid *et al.*, 2018; Park *et al.*, 2021). Interspecific RNA transfer offers many future possibilities (Johnson and Axtell, 2019) and will probably remain a topic of intense research in the foreseeable future.

### Cues that give away the host location

Because *Cuscuta* displays a high resistance towards most commercially available pesticides [with the exception of the highly toxic dinitroaniline herbicides (Dawson, 1990)], the parasite is regarded widely as a difficult-to-control pest (Nadler-Hassar and Rubin, 2003; Goldwasser *et al.*, 2012). Therefore, the search for effective pest control measures has focused, among other things, on how *Cuscuta* finds its potential hosts. For the parasite, host detection is crucial, particularly at the seedling stage because the seed offers only limited resources. However, the triggering of host penetration and invasive growth by the haustorium also rely on external cues. Those signals help time the haustorial induction and are important for the success of the parasite. In the late 19th century, it was already speculated that dodders may be able to sense and differentiate between different hosts (Peirce, 1893). The notion of *Cuscuta* displaying chemotropism was reiterated a number of times throughout the last century (Bünning and Kautt, 1956; Kelly, 1992). However, the actual attractants that cause the chemotropism were not discovered until Runyon *et al.* (2006) identified several volatile organic compounds that could attract *Cuscuta pentagona*. The authors used a smart experimental setup to show that the parasite reacts to volatiles and identified a range of monoterpenes as the active components in the attraction process (Runyon *et al.*, 2006). Although the sensing of chemicals has not been shown for other species and has since not led to any practical approaches to disorient the parasite in the field, this discovery can be considered a milestone in *Cuscuta* research (Fig. 3).

Another cue that *Cuscuta* appears to use avidly is light. Unlike most normal plants that display shade avoidance, *Cuscuta* is attracted by the canopy shade of their hosts. Canopy shade is characterized by a spectral composition depleted in red (R) and blue wavelengths due to absorption by photosynthetic pigments in the host foliage, but with a higher relative amount of far-red (FR) light. Therefore, the observation that light with a low R:FR ratio (indicating shade, and therefore host presence) attracts seedlings and shoots of *Cuscuta* and induces photomorphogenic changes (Lane and Kasperbauer, 1965; Orr *et al.*, 1996) was a significant advance in the understanding how parasites locate their hosts using a known molecular aide: phytochrome. While the tropism towards a host is governed by the R:FR ratio, the coiling around a host stem may be also induced by blue light. With modern LED technology that allows a much better resolution of light doses and light quality, Smith *et al.* (2021) showed recently that the parasite is even able to differentiate between minute differences in the wavelength signatures and can thereby derive information not only on host proximity but also on host architecture from the R:FR ratios within the white light. Whether *Cuscuta* employs the same changes in hormone concentrations (especially auxin) as found during the shade avoidance of autotrophic plants has, to our knowledge, not been investigated. Similarly, it is not yet known if the signaling cascades downstream of phytochrome perception are the same as in non-parasites.

Interestingly, haustorium development was also shown to be regulated by the light quality (Tada *et al.*, 1996) (Fig. 3). It was demonstrated that FR induction of haustorium formation is a result of the action of phytochrome because the FR effect can be reversed by subsequently given red light (Furuhashi *et al.*, 1997). This has been exploited in several studies to induce and analyze haustorium development (Olsen *et al.*, 2016; Bawin *et al.*, 2022) and to develop transformation protocols (Lachner *et al.*, 2020). A recent paper reported the differential regulation of genes from the auxin signaling pathway upon treatment with light of a low R:FR ratio (Pan *et al.*, 2022). This supports the notion that at least some of the downstream cascades that phytochrome signaling normally induces are also visible in the light-controlled infection process. However, in contrast to the light-mediated directional growth of *Cuscuta*, light-mediated haustoriogenesis requires an additional touch or tactile stimulus (Tada *et al.*, 1996). The induction of haustoria after a FR treatment was only observed on the side of the stem facing the host or by the part of the stem touching an inert surface such as a glass or acrylic dish (Tada *et al.*, 1996; Furuhashi *et al.*, 1997). Cytokinin has been reported to activate haustoriogenesis without FR light and is therefore anticipated to be involved in the signal transduction downstream of the initial light and contact signals (Ramasubramanian *et al.*, 1988; Haidar *et al.*, 1998; Furuhashi *et al.*, 2011). It remains unknown how these factors are integrated in the decision of whether to commit to haustoriogenesis or not.

### In search for a cure against *Cuscuta* attacks

As described before, *Cuscuta* spp. infect a broad spectrum of host plants, with a preference for dicot host species. Although the parasite modifies and even degrades the host’s cell walls (Nagar *et al.*, 1984; Olsen *et al.*, 2016), attacks go seemingly unrecognized by the attacked plants. However, a few noteworthy exceptions were found in the genus *Solanum* according to a report published in the late 1980s (Ihl *et al.*, 1988) (Fig. 3). During the following two decades, a number of studies comparing the interaction of different tomato cultivars with different *Cuscuta* species were published (e.g. Löffler *et al.*, 1996; Goldwasser *et al.*, 2001; Miranda-Sazo, 2003). These revealed a considerable variation in the outcome of a parasitic attack depending on which *Cuscuta* species was involved. Generally, *C. reflexa* was found to be most strongly deterred by the physical and physiological barriers implemented by tomato hosts, while members of the subgenus *Grammica* were found to be less affected (summarized by Lanini and Kogan, 2005). Several publications have taken advantage of this tolerance of tomato lines to some *Cuscuta* species to investgate aspects of the host–parasite relationship (Jiang *et al.*, 2013; Ranjan *et al.*, 2014; Hegenauer et al., 2016, 2020; Krause *et al.*, 2018) (Fig. 3).

Phenotypically, the defense reaction by tomato plants against *C. reflexa* is clearly visible as a hypersensitive response on the tomato stems at the sites of attempted haustorium penetration (Ihl *et al.*, 1988; Sahm *et al.*, 1995; Runyon *et al.*, 2010). In the late attachment phase, ~3–5 d after the parasitic pre-haustorium has formed, epidermal host cells at the contact sites elongate and rupture (Werner *et al.*, 2001; Albert *et al.*, 2004). Living cells of the tissue below secrete phenylpropanoids and show an increased activity of peroxidases, which are important for linking phenylpropanoids with other cell wall components such as cellulose fibers, pectin, or proteins. Together with synthesized lignins and long-chained di-fatty acids, these enzymes facilitate the cross-linking of cell wall compounds to the formation of a suberin-like structure. This barrier fends off the *C. reflexa* haustorium before it can establish any feeding connections (Kaiser *et al.*, 2015). The observed resistance phenomenon of selected tomato species against a subset of *Cuscuta* species hints at a mechanism comparable with ‘race-specific resistance’ as described for gene for gene interactions.

The molecular resistance mechanism of *Solanum lycopersicum* against *C. reflexa* is not yet completely understood. The wild tomato relative *S. pennellii* is fully susceptible and this allowed for exploration of the genetic resources to understand the underlying mechanisms. To this end, the collection of *S. lycopersicum×S. pennellii* introgression lines (Eshed and Zamir, 1995; Chitwood *et al.*, 2013) has been very useful in the attempt to identify resistance gene candidates. Regions conferring susceptibility to *C. reflexa* were discovered on chromosomes 1, 2, 6, and 8 (Hegenauer *et al.*, 2016; Krause *et al.*, 2018). One tomato gene identified by this approach encodes a leucine-rich repeat receptor protein (LRR-RP)—*Cuscuta* receptor 1 (CuRe1)—which plays an important role in the recognition of *C. reflexa* and in the induction of defense-related responses (Hegenauer *et al.*, 2016) (Fig. 3A). CuRe1 is a plasma membrane-bound receptor lacking an intracellular kinase domain. With its extracellular LRR domain, CuRe1 recognizes a 116 amino acid long, glycine-rich protein of the *C. reflexa* cell wall (CrGRP) as a molecular pattern that identifies *Cuscuta* as an attacking pathogen (Hegenauer *et al.*, 2020). As a minimal epitope, the 21 amino acid long peptide Crip21 binds and activates tomato CuRe1 and specifically induces classical PAMP (pathogen-associated molecular pattern)-triggered immune responses such as the elevation of reactive oxygen species (ROS burst) or the production of the stress-related phytohormone ethylene (Fürst *et al.*, 2016; Hegenauer *et al.*, 2016). CuRe1 also contributes to host plant resistance and supports a restriction of *C. reflexa* growth when transformed into usually susceptible hosts. However, *CuRe1* seems not to be sufficient for resistance alone, but may be supplemented by additional molecular factors, probably additional receptors, that appear to be critical for a complete tomato resistance against *C. reflexa* (Krause *et al.*, 2018; Jhu *et al.*, 2022). While CuRe1 is a plasma membrane-bound receptor and represents an essential component of pattern-triggered immunity (PTI), a CC–NBS–LRR protein that seems to be part of the effector-triggered immunity (ETI) and is involved in lignin-based resistance of tomato against *C. campestris* has been identified (Jhu *et al.*, 2022). This intracellular receptor, named CuRLR1 (*Cuscuta* Receptor for Lignin-based Resistance 1), whose ligand is as yet unknown, probably acts as an important key component together with at least two transcription factors and contributes to host resistance against *C. campestris*. Since deciphering the molecular mechanisms of host plant resistance against parasitic *Cuscuta* could be the key to create resistant crops against parasitic plant attacks, this line of research is assumed to continue well into the future.

### The missing link for becoming a parasitic plant model organism: genetic modification

The idea of genetically modifying *Cuscuta* and creating transgenic parasites has already been evaluated ~20–25 years ago. A central step in genetic modification of plants is via tissue culture, allowing successfully transformed cells to be propagated and regenerated into a whole plant. The first reports of successful formation of callus and regeneration via embryogenesis, as well as subsequent maintenance of the formed shoots without the need for a host was reported for *Cuscuta trifolii* (Bakos et al.,1995, 2000). However, callus-based regeneration failed after *Agrobacterium tumefaciens*-mediated transformation of the tissue (Borsics *et al.*, 2002), limiting the applicability of this approach. Similar attempts were reported for *C. reflexa* (Srivastava and Dwivedi, 2001) and *C. europaea* (Svubova and Blehova, 2013) (Fig. 3A), but were also unsuccessful. That transformed cells are recalcitrant to regeneration was also shown by Lachner *et al.* (2020) who could observe expression of transgenically introduced fluorophores over many weeks, but without being able to induce propagation of the transformed cells or formation of a callus.

Host-induced gene silencing (HIGS) as a method of RNAi using transgenic host plants that express siRNAs has been tried successfully on *C. pentagona* (Alakonya *et al.*, 2012) and *C. campestris* (Jhu *et al.*, 2021). However, this method is very labor-intensive because a new transgenic host has to be created for every gene of interest individually. Virus-induced gene silencing (VIGS) also utilizes the RNAi pathway for silencing but is a slightly less elaborate approach. VIGS-based transformation has been recently reported for *C. campestris* (Dyer *et al.*, 2021, Preprint), but at present this method still needs to be optimized to enable the expression of the transgene also in newly developed shoots further away from the infection site on the virus-carrying host. In terms of inheritable transgenes, there is still no reportable breakthrough, making this one of the biggest bottlenecks in *Cuscuta* research.

## Conclusion

Despite many differences from their autotrophic hosts, it has not been possible to find a trait that would qualify as a promising starting point for developing an efficient remedy against *Cuscuta*. Given some of the reported spreads of the parasite into new habitats (Masanga *et al.*, 2021), the search for an effective remedy against *Cuscuta* infection is therefore still highly relevant and timely. At present, the inability to genetically modify the parasite is still a major bottleneck for making breakthrough advances in this respect. Protocols for stable transformation of *Cuscuta* are presently being sought intensively, and it is very likely that it will only be a matter of time until this will be achieved. On the other hand, we are seeing an increase in approaches where *Cuscuta* is used as a model to illuminate basic biological processes in plant cells from a different angle. Examples are interspecific connectivity and plasmodesmata development, virus transfer and transport processes in general, self- and non-self-distinction, or evolutionary processes in the genomes, to name a few. It has been recently shown that *Cuscuta* can be propagated entirely without a living host using an artificial system for support (Bernal-Galeano *et al.*, 2022). This system will allow a tighter control over the exposure of the feeding interface to external factors and will add to the list of topics that *Cuscuta* research can be useful for. These current developments benefit from the comprehensive knowledge that has been collected on *Cuscuta* over the last 100 years and more.
